# Rules for Orthopedic Practice

**Published:** 1898-04

**Authors:** 


### RULES FOR ORTHOPEDIC PRACTICE.

1. Discard the idea of rheumatism of a single joint in children (especially in the hip) unless it is positively proved by acute symptoms. Tuberculous disease is much more probable, and treatment for the graver condition will frequently abort threatened joint-destruction.

2. Any child may have local tuberculosis of a joint, no matter what its ancestry; heredity signifies only degree of resistance.

3. Do not attribute to "habit," a persistent limb. An inflammatory or paralytic cause is more probable.

4. All persistently fretful infants (especially those that cry when moved) should be carefully examined for evidences of spinal spondylitis. Older children, with stubborn irritation of the lung, stomach or intestine, should always be critically examined. Early diagnosis and treatment will accomplish excellent results.

5. In lateral curvature of the spine the indiscriminate and unskillful use of mechanical appliances does harm, while to neglect these means of fixation in spinal caries is ruinous.

6. To "let alone" a curable congenital bone-deformity after the first week of life is to lose the golden opportunity for cure.

